# Vinculin Interacts with the *Chlamydia* Effector TarP Via a Tripartite Vinculin Binding Domain to Mediate Actin Recruitment and Assembly at the Plasma Membrane

**DOI:** 10.3389/fcimb.2015.00088

**Published:** 2015-11-30

**Authors:** Tristan R. Thwaites, Antonio T. Pedrosa, Thomas P. Peacock, Rey A. Carabeo

**Affiliations:** ^1^Programme in Microbiology, Institute of Medical Sciences, University of AberdeenAberdeen, UK; ^2^Medical Research Council Centre for Molecular Bacteriology and Infection, Imperial College LondonLondon, UK; ^3^School of Molecular Biosciences, Washington State UniversityPullman, WA, USA

**Keywords:** *Chlamydia*, signal transduction, vinculin, actin cytoskeleton, type III effectors

## Abstract

The mammalian protein vinculin is often a target of bacterial pathogens to subvert locally host cell actin dynamics. In *Chlamydia* infection, vinculin has been implicated in RNA interference screens, but the molecular basis for vinculin requirement has not been characterized. In this report, we show that vinculin was involved in the actin recruitment and F-actin assembly at the plasma membrane to facilitate invasion. Vinculin was recruited to the plasma membrane via its interaction with a specific tripartite motif within TarP that resembles the vinculin-binding domain (VBD) found in the Shigella invasion factor IpaA. The TarP-mediated plasma membrane recruitment of vinculin resulted in the localized recruitment of actin. *In vitro* pulldown assays for protein-protein interaction and imaging-based evaluation of recruitment to the plasma membrane demonstrated the essential role of the vinculin-binding site 1 (VBS1), and the dispensability of VBS2 and VBS3. As further support for the functionality of VBD-vinculin interaction, VBD-mediated actin recruitment required vinculin. Interestingly, while both vinculin and the focal adhesion kinase (FAK) colocalized at the sites of adhesion, the recruitment of one was independent of the other; and the actin recruitment function of the VBD/vinculin signaling axis was independent of the LD/FAK pathway.

## Introduction

In mammalian cells, the actin-binding protein vinculin associates with the cytoplasmic face of focal adhesions to facilitate linkage of integrin molecules to the actin cytoskeleton (Critchley et al., 1999). Structurally, vinculin is composed of three major domains: an N-terminal head (Vh), a flexible proline-rich hinge region, and a C-terminal tail domain (Vt). Intramolecular associations between the head and tail domains constrain vinculin to an inactive state (Johnson and Craig, 1995). Vinculin activation requires the interaction with another focal adhesion protein, talin, which is mechanically stretched upon local application of tensile force at focal adhesions to expose vinculin binding sites (VBS) (Fillingham et al., 2005; Humphries et al., 2007; del Rio et al., 2009; Grashoff et al., 2010). Talin initially associates with Vh domain 1 (Vh1) of inactive vinculin through hydrophobic surface interactions (Gingras et al., 2005). Switching to an open active conformation allows for complete binding of vinculin to talin (Case et al., 2015). In its active form, vinculin is capable of direct interaction with Arp2/3, actin, phosphatidylinositol (4,5)-bisphosphate (PIP2), and paxillin (Turner et al., 1990; Hüttelmaier et al., 1998; DeMali et al., 2002). Thus, the binding of vinculin to talin can facilitate a localized increase in the number actin filaments and the maturation of focal adhesions.

Unsurprisingly, a number of bacterial pathogens have been shown to employ vinculin to facilitate infection of host cells and dissemination within tissues. Focal adhesion proteins including talin and vinculin have been found to accumulate within *Salmonella*-induced membrane ruffles (Finlay et al., 1991). More recently, the *Rickettsia* cell surface antigen, Sca4, and the *Shigella flexneri* effector protein, IpaA, have been shown to bind and activate vinculin (Tran Van Nhieu et al., 1997; Tran Van Nhieu and Izard, 2007; Park et al., 2011a). Both Sca4 and IpaA connect to host F-actin by functioning as talin mimics to bind and activate vinculin (Hamiaux et al., 2006; Park et al., 2011a,b). In each case, the virulence factors harbor at least two VBSs.

Signaling required for *Chlamydia* invasion is initiated by the attachment of the infectious elementary body (EB) to the host cell surface, where it either engages a number of host cell receptors and/or activate its type III secretion system (T3SS) to translocate the effector TarP (Clifton et al., 2005; Elwell et al., 2008; Lane et al., 2008; Mehlitz and Rudel, 2013). It is unclear if an individual EB simultaneously activates all of the pathways experimentally implicated or if the infecting organism display preference for one specific pathway depending on the context of infection, including cell type and two- or three-dimensional culture configuration. In addition, the functional relationships between the characterized pathways have not been fully elucidated. Regardless, the net result is the robust recruitment of actin at the sites of chlamydial adhesion, leading to the engulfment and internalization of the EB (Carabeo et al., 2002; Subtil et al., 2004; Balañá et al., 2005).

TarP is a chlamydial virulence factor that functions to recruit signaling molecules at the plasma membrane via a number of domains that resemble mammalian signaling motifs (Lane et al., 2008; Carabeo, 2011). We have previously identified the signaling function of the N-terminal phosphodomain of TarP of *Chlamydia trachomatis* by interacting with Src homology 3 (SH3)-domain containing adapter proteins to recruit two forms of Rac GTPases to induce an Arp2/3 complex-mediated nucleation of actin polymerization at the sites of invasion (Lane et al., 2008). Also, we have recently reported on the role in actin recruitment of the FAK-binding motif (LD) present in TarP (Thwaites et al., 2014). Jewett et al. also functionally characterized the role of the TarP actin-binding domains in actin nucleation (Jewett et al., 2006). The multiple mechanisms of actin recruitment and polymerization beg the question of functional interaction. Jewett et al. demonstrated a cooperation between Arp2/3- and direct TarP-mediated actin nucleation processes (Jiwani et al., 2012).

To date, the question of whether vinculin plays a role in *Chlamydia* invasion remains largely unknown, but RNA interference screens have identified vinculin being necessary for *C. trachomatis* L2 infection *in vitro* (Elwell et al., 2008; Gurumurthy et al., 2010). Both screens assessed inclusion formation, and not chlamydial invasion specifically. In this study, we show that vinculin is necessary for invasion, and is recruited by *Chlamydia* at the sites of adhesion in a FAK-independent, but TarP-dependent fashion. Three putative VBSs located at the C-terminus of TarP of *Chlamydia caviae* comprising the vinculin binding domain (VBD) was functionally characterized. Examination of other TarP orthologs revealed conservation amongst the chlamydial species, underscoring its biological importance. VBD was the sole domain in TarP that interacted with vinculin, and the each VBS displayed varying levels of vinculin binding *in vitro*. The VBD motif is competent in signaling to mediate localized actin recruitment at the plasma membrane in a vinculin-dependent, but FAK-independent manner. Furthermore, the presence of the proximal FAK-binding LD motif was not required. Enhancement in the frequency of actin recruitment was not observed in the presence of the LD motif.

## Materials and methods

### Reagents, cell lines, and organisms

Anti-HA.11 clone 16B12 mAb was purchased from Covance; anti-FAK (phospho Y397) pAb and anti-vinculin mAb were from Abcam. Anti-rabbit or anti-mouse IgG secondary antibodies, either Alexa Fluor 488 or 594 were purchased from Invitrogen. Phalloidin conjugated to Alexa Fluor dye was purchased from Invitrogen. Cos7 (ATCC CRL-1651) and HeLa 229 (ATCC CCL-2.1) were routinely grown in DMEM supplemented with 10% FBS, 2 mM L-glutamine, and 10 μg/ml gentamicin. Subcultivation was at 1:4 ratio. The cells were used at passage < 15. FAK^−∕−^ mouse embryo fibroblasts (MEFs; CRL-2644) and matched FAK^+∕+^ cells (CRL-2645) were purchased from LGC standards. *vcl*^−∕−^ and matched *vcl*^+∕+^ MEFs (Marg et al., 2010) were generously provided by Dr. Wolfgang Ziegler (Hannover Medial School). All MEFs were cultured in DMEM + 10% FBS and subcultured at 1:4 ratio. Cultured cells were grown in a humidified 5% CO2 incubator at 37°C. *C. caviae* strain GPIC was propagated in HeLa cells grown in DMEM + 10% FBS supplemented with gentamicin (10 μg/ml). Harvest of elementary bodies was by discontinuous density gradient centrifugation in Renografin (Bracco Diagnostics), as previously described (Caldwell et al., 1981).

### Cloning

The VBD (VBS1-*LLEAARNTTTMLSKTLSKV*; Thr^714^-Ser^880^), VBS1^mut^-2-3 (Mutant VBS1-*LLE**SS**RNTTTM**SS**KT**S**S**S**V*; Thr^714^-Ser^880^), ΔVBS1 (Thr^714^-Arg^832^), ΔVBS2,3 (Leu^837^-Ser^880^), ΔVBS1,2 (Thr^714^-Thr^784^), the LD (Ser^640^-Pro^740^), and the LD-VBD (Ser^640^-Ser^880^) fragments were amplified from the *C. caviae* GPIC TarP clone (Clifton et al., 2005) using primer pairs indicated in Table 1. Primers were engineered with KpnI linkers. GPIC TarP deleted of the proline rich domain (PRD; Ile^355^-Val^399^) to aid solubility during overexpression was generated by inverse PCR from a GPIC TarP clone (Clifton et al., 2005) using primer pair 13–14. Deletion of the VBS3 (Leu^746^-Ala^764^) or VBS2 (IIe^806^-IIe^824^) was also facilitated by inverse PCR using primer pairs 31–32, 9–10 and 11–12, respectively. The TarP deletion variant (Met^1^-Thr^714^) was generated from GPIC TarP clones (ΔPRD) using primer pairs 9–10 and 11–12 engineered with Kpn1 linkers for fusion with the TirM derivative of the TirMC plasmid (pKC87). This plasmid has been previously described by Campellone et al. (2004) and was generously provided by Professor John Leong (Tufts University). Construction of TirM involved PCR amplification of part of TirMC that contained amino acids 1–66, which encodes the essential membrane targeting domain of the Newcastle Disease Virus HN surface protein, the hemagglutinin (HA) epitope tag and Tir amino acids 260–395. The “C” region of TirMC was excluded, and in its place a unique Kpn1 site was introduced at the 3′ end of the TirM open-reading frame using primer pair 1–2. This facilitated the cloning of TarP fragments to create TirM-TarP fusion derivatives. Blunt-end TirM PCR products were cloned directionally by TOPO® Cloning into a pENTR™/D-TOPO® entry vector (Invitrogen) to create pENTR-TirM. The products generated for TarP-FL, its deletion variants, and isolated regions of interest were digested with KpnI and subcloned into linearized pENTR-TirM to generate translational fusions with TirM at the N-terminus. Expression clones were generated through LR recombination between entry clone and pcDNA-Dest40 (Invitrogen), which had the CMV promoter to drive expression in mammalian cells. All constructs were verified by DNA sequencing.

**Table 1:** **Primers used for PCR amplification in this study**.

**Primer ID**	**Primer name**	**Sequence (5′-3′)**
1	TirM Fwd	CACCATGGGCTTAGGAAATGATGAAAGGGAACGG
2	TirM Rev	AGTATTGGTACCCCTGTTCTGCCGGCTG
3	PD 1xR Fwd	GCAGATAGGTACCGTATGACTTCAGAAAGCTCAGAAACT
4	PD 1xR Rev	GCGGATGGTACCTTAGTAGGAGGAGCCTCTTAGA
5	VBS1 Fwd	CATATCAGGTACCGTATGTTGGATAGTGCAGATACA
6	VBS1 Rev	ATAGTGGATGGTACCTTAGGAGTGTCTTTGAGG
7	VBS3 Fwd	GAGGGCAGGTACCGTATGACTAGCAGTTCTGCA
8	VBS3 Rev	GATGCGGATGGTACCTTAACAGGTTGCTGTCTC
9	ΔVBS3 Fwd	TCTACGACAAGCACTACGGTAAGC
10	ΔVBS3 Rev	GTCAGGGGAAGGTCCAGG
11	ΔVBS2 Fwd	GAAAAAGGCGCAAGATTGCAA
12	ΔVBS2 Rev	TCCTGAAGGAGACCCTCC
13	ΔVBS1 Fwd	GAGGGCAGGTACCGTATGACTAGCAGTTCTGCA
14	ΔVBS1 Rev	GCTGCGGATGGTACCTTATCTTGCGCCTTTTTC
15	VBS1(mut)-2-3 Fwd	GAGGGCAGGTACCGTATGACTAGCAGTTCTGCA
16	VBS1(mut)-2-3 Rev	TAATATTATTTTGGTACCTTACCCCGTCACAGAAG-AAGAGGTTTTTGAAGACATTGTTGTTGTATTTCTAGAAGATTC
17	VBD Fwd	GAGGGCAGGTACCGTATGACTAGCAGTTCTGCA
18	VBD Rev	ATAGTGGATGGTACCTTAGGAGTGTCTTTGAGG
19	LD Fwd	GTAAATAGGTACCGTATGTCTTCTGAATCACGAGCC
20	LD Rev	GTCGCTTGTGGTACCTTATTTATCTCCCCCTGTACC
21	pHom LD Fwd	GTACGTGACTAGTTCTTCTGAATCACGAGCC
22	pHom LD Rev	GCTTGCACTAGTAGGAGTCGTTCTTTCTGC
23	LD-VBD Fwd	GTAAATAGGTACCGTATGTCTTCTGAATCACGAGCC
24	LD-VBD Rev	ATAGTGGATGGTACCTTAGGAGTGTCTTTGAGG
25	mutLD Fwd	AAAATAATAGGTACCGTATGAGTGCTGCAGGTGGTGA-GGGCGCAGAAGGAGCCGAGCATGCAGCACCACAGGCA
26	mutLD Rev	GGCGCCGCGGGTACCTTAAGGAGTCGTTCTTTCTGC
27	TarP^1−639^ Fwd	GCCCTCAGGTACCGTATGACTAGTCCTATT
28	TarP^1−639^ Rev	GCAGCTAGGTACCGTATGAAGCCTACAGTATTGTTA
29	TarP^1−714^ Fwd	GCCCTCAGGTACCGTATGACTAGTCCTATT
30	TarP^1−714^ Rev	GTCGCTTGTGGTACCTTATTTATCTCCCCCTGTACC
31	TarP PRD Fwd	AATGTTACTGGAGGGACAACGACG
32	TarP PRD Rev	TCCTTTCAAACCACCGACATCACC
33	TarP FL Fwd	GCCCTCAGGTACCGTATGACTAGTCCTATT
34	TarP FL Rev	TCTTATGGTACCGGAGTGTCTTTGAGGTGG

### Transfection and indirect confocal microscopy

Cos7 were transfected with full-length TarP, the VBD, VBD derivatives, the LD, or the LD-VBD using Lipofectamine 2000 (Invitrogen) as described by the manufacturer. At 32 h post-transfection, the cells were fixed by adding 4% paraformaldehyde. Antibodies diluted in 1 × PBS to their respective working concentrations (anti-HA.11 clone 16B12 1:500; anti-vinculin 1:250) were added to fixed cells and incubated 37°C for 1 h. Actin was detected with phalloidin (1:250 dilution) conjugated to Alexa Fluor dye. Coverslips were mounted onto glass slides using mowiol. Sample visualization was performed at RT on a laser scanning microscope (LSM 510; Carl Zeiss) using an oil-immersion PlanApochromat 63×/1.40 NA differential interference contrast. Images were processed using NIH ImageJ freeware (Schneider et al., 2012) or Adode Photoshop CS5. Pixel intensities were measured using ImageJ freeware. Incidences of colocalization were assessed visually in a single-blind experiment by someone not involved with image acquisition. For the generation of heat maps of pixel intensity, images for each cell population were taken with identical exposure times and on the same day to allow accurate comparison of fluorescence intensities between images. Images were exported into NIH ImageJ for analysis.

### Co-immunoprecipitation and western blot analysis

Briefly, Cos7 cells from five 6-well culture plates expressing HA-tagged proteins were washed once with phosphate-buffered saline (PBS) at room temperature and then lysed with 0.5 ml ice-cold IP lysis buffer (Pierce). Cells were scraped, transferred into a 1.5 ml microcentrifuge, and vortexed briefly. Lysis was continued on ice for 10 min, after which the cell debris was pelleted at 15,000 rpm. in a microcentrifuge. Lysates were incubated with anti-HA magnetic beads (Pierce) at 4°C with rocking as described by the manufacturer. The beads were then washed twice with 0.3 ml 0.05% TBS-T (Tris-buffered saline supplemented with Tween) and then eluted with two 50-μl washes of 2 mg/ml HA-peptide (Pierce) in TBS. The eluted samples were mixed with Laemmli buffer and boiled prior to gel electrophoresis. Proteins were probed with anti-vinculin (1:1000 dilution) (Abcam) or anti-HA (1:1000 dilution) (Covance) antibodies followed by the addition of HRP-conjugated secondary antibody at 1:1000 dilution (Cell Signaling Technology).

### Bacterial infection

For EPEC, bacterial cultures were primed for infection as previously described (Wong et al., 2012) with the following modification. A 1:500 dilution of overnight culture was used to prime the bacterial culture for infection. Cos7, *vcl*^+∕+^ and *vcl*^−∕−^ cells were grown in 24-well cell culture plates to 80% confluence. The cells were transfected with 100 ng plasmid DNA. Transfected cells were incubated at 37°C in a humidified incubator for 32 h prior to infection. Cells were infected as previously described (Campellone et al., 2004). The infection was allowed to proceed for 4 h in the presence of gentamicin (200 mg/ml) (Gibco) after the first hour. Δ*tir* EPEC were visualized using DAPI diluted at 1:1000. For *Chlamydia*, HeLa, *vcl*^−∕−^*, vcl*^+∕+^, FAK^−∕−^, and FAK^+∕+^ cells were infected as previously described (Thwaites et al., 2014) with the following modifications. Cells were infected at a multiplicity of infection (MOI) of 50 for *C. caviae GPIC* and spun (1500 rpm for 3 min) at 4°C (HeLa) or room temperature (RT; *vcl*^−∕−^*, vcl*^+∕+^, FAK^−∕−^ and FAK^+∕+^) to allow maximum chlamydial adherence to target cells.

### Invasion assay

Efficiency of *C. caviae* strain GPIC invasion was performed using a double staining and fixation protocol as described previously (Carabeo et al., 2002; Subtil et al., 2004) with the following modifications. *vcl*^−∕−^ and *vcl*^+∕+^ cells were infected *C. caviae* at MOI of 1 and spun at 1500 rpm for 3 min at RT to synchronize infection. Unattached EBs were removed by washing cells three times with room temperature (RT) IMDM media. Pre-warmed IMDM media was added and the infection was allowed to proceed at 37°C for the appropriate time p.i. Coverslips were washed three times in PBS and fixed with 4% paraformaldehyde for 15 min. Extracellular EBs were labeled with a *C. trachomatis* anti-LPS 1:750 (Abcam) primary and Alexa Fluor 488 conjugated secondary antibody (Invitrogen). Cells were permeabilised with 0.25% Triton X-100 for 5 min and washed 3× with PBS before a secondary fixation step with 4% PFA for 15 min. Total EBs were labeled with an anti-*Chlamydia* antibody as the primary antibody at 1:750 dilution (Abcam) and Alexa Fluor 594-conjugated secondary antibody (Invitrogen).

### Bioinformatics

All figures involving the alignment or phylogenetic analysis of nucleic acid sequence were generated using ClustalW. Parameters for alignments shown are indicated within the figure legends describing the alignment. Helical wheel representations were generated using PROTEAN from DNASTAR inc.

### Statistical analysis

All statistical analyses were run using the program StatPlus. Unless stated otherwise, statistical differences were tested using One-way analysis of variance (ANOVA) and Tukey's *post-hoc* test. Box and Whisker Plots show range (statistical outliers excluded), the lower and upper quartile values (box) with median (horizontal line) and mean (diamond). Whiskers extend from each end of the box to the 10th and 90th percentiles. Where stated, the significance level α was set to 0.05 or 0.01. All *p*-values ≤ α were considered statistically significant.

## Results

### *Chlamydia* requires vinculin for invasion

**Figure 1:**
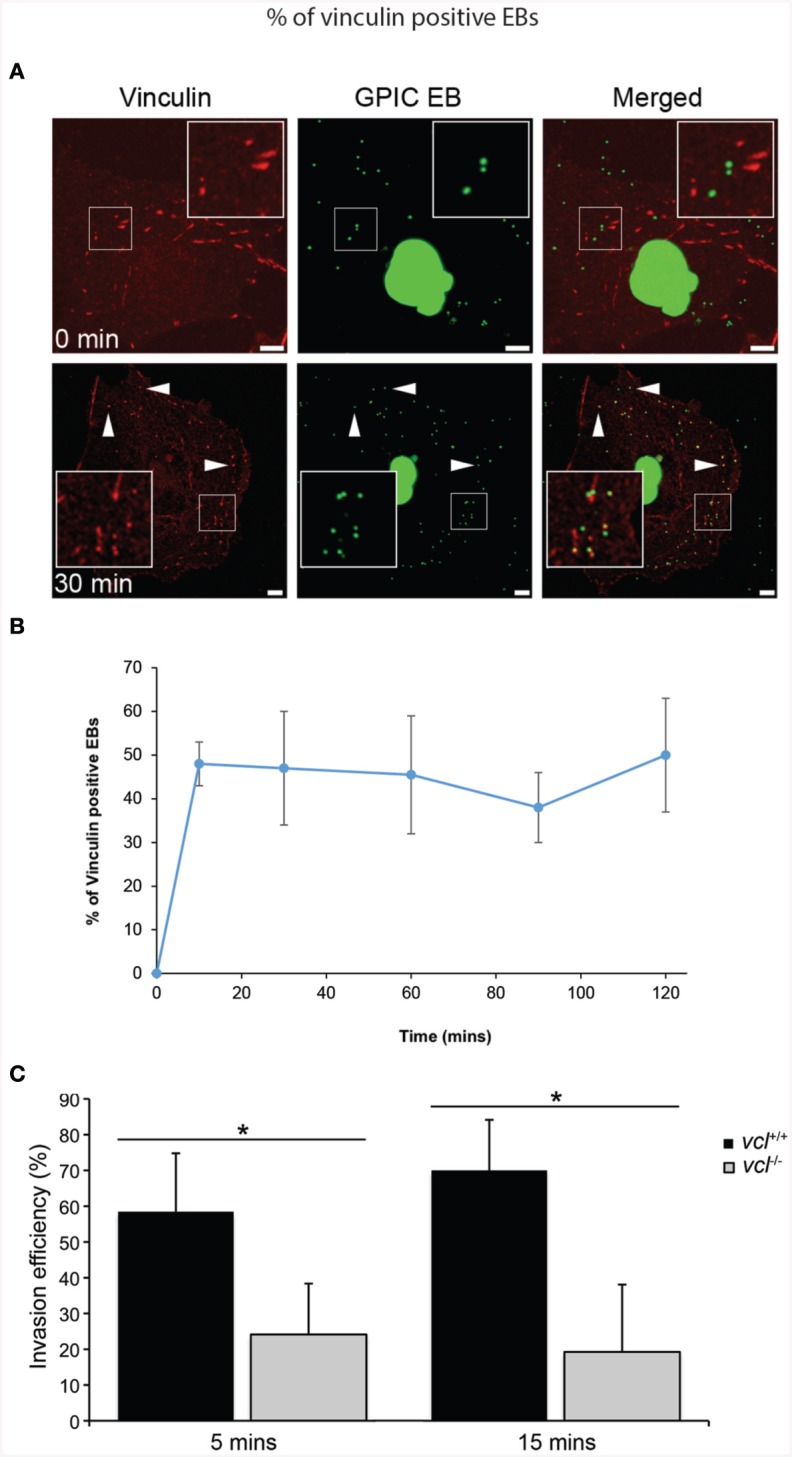
*****Chlamydia*** recruits vinculin to sites of invasion**. **(A)** *C. caviae* (GPIC) elementary bodies (EBs) were added to Cos7 cells at 4°C, and shifted to 37°C by the addition of pre-warmed media to synchronize invasion. The infection was allowed to proceed for 0, 10, 30, 60, 90, or 120 min at which time the cells were fixed and process for immunofluorescence staining and microscopy. Cells were stained with an anti-vinculin antibody (red) and *Chlamydiae* were visualized by DAPI (green). Note the increase in the number of co-localization events from 0 to 30 min. Images for the other time points are shown in Figure S1. Scale bars: 5 μm. White arrowheads indicate vinculin colocalizing with GPIC EBs. **(B)** Colocalization frequency per cell was calculated for each time point up to 120 min post-infection, with at least 50 cells analyzed per time point. There is a rapid increase of vinculin recruitment by 10 min p.i., which was sustained up to 120 min p.i. **(C)** *vcl*^+∕+^ and *vcl*^−∕−^ cells were infected with *C. caviae* (GPIC). Infection was allowed to proceed up to 15 min post-temperature shift. Cells were fixed and processed for the invasion assay as detailed in the Materials and Methods Section. GPIC invasion was evaluated in *vcl*^+∕+^ and *vcl*^−∕−^ cells. *vcl*^−∕−^ cells were less able than wild-type to support GPIC invasion at 5 or 15 min post-infection (p.i.). Data are from a minimum of 150 cells from three independent experiments, and expressed as means ± SD. Asterisk and bar indicates statistical significance between specific groups (One-way ANOVA, Tukey's *post-hoc* test, *P* < 0.008).

Vinculin has been shown to be a target of a number of microbial pathogens as they take advantage of its ability to mobilize actin. Given previous findings demonstrating the involvement of various focal adhesion components during Chlamydial infection (Coombes and Mahony, 2002; Elwell et al., 2008; Gurumurthy et al., 2010), we sought to determine in greater detail the involvement of vinculin in *Chlamydia* invasion. Using HeLa cells, vinculin recruitment to the sites of invasion was evaluated. HeLa cells were infected with *C. caviae* GPIC and vinculin recruitment monitored at 0, 10, 30, 60, 90, and 120 min after synchronization by temperature-shift to 37°C. Cells were immediately fixed with freshly prepared 4% paraformaldehyde at the designated time points. As shown in Figure 1A, vinculin was observed to be strongly concentrated around EBs as early as 10 min post-infection (p.i.). From confocal microscopy images of infected cell monolayers, we quantified the levels of colocalization of EBs to vinculin. The graph in Figure 1B shows recruitment at 10 min p.i., and that the steady state levels remained elevated until at least 120 min p.i.

To address the chlamydial requirement for vinculin, we exploited mouse embryo fibroblasts (MEFs) ablated for the *vcl* gene (*vcl*^−∕−^). To avoid functional redundancy in signaling to the actin remodeling machinery, *C. caviae* GPIC was chosen because it lacked the N-terminal phosphodomain previously shown to promote Sos1- and Vav2-mediated actin recruitment in *C. trachomatis* (Lane et al., 2008). The absence of the phosphodomain in the *C. caviae* TarP ortholog should enable us to definitively assign signaling pathways to the motifs in the C-terminal half of TarP. Invasion assays in wild type (WT) and *vcl*^−∕−^ MEFs by *C. caviae* GPIC EBs were performed and quantified as previously described (Thwaites et al., 2014). Invasion was monitored at 5 and 15 min post-infection (p.i.), and data was expressed as invasion efficiency per cell. As shown in Figure 1C, the invasion efficiencies of *vcl*^−∕−^ MEFs were significantly different from the *vcl*^+∕+^ MEFs at both time points post infection (p.i.) (*p* < 0.005). The incomplete inhibition of invasion in vinculin-depleted cells was likely to be related to redundant invasion pathways, including the FAK-dependent pathway that signals from the LD domain of TarP (Thwaites et al., 2014). Overall, the highly localized recruitment of vinculin to the sites of entry, and the requirement for efficient invasion suggest that vinculin has an essential role in the infection of cultured cells by *C. caviae* GPIC.

### Identification of the vinculin binding domain in the chlamydial effector TarP

**Figure 2:**
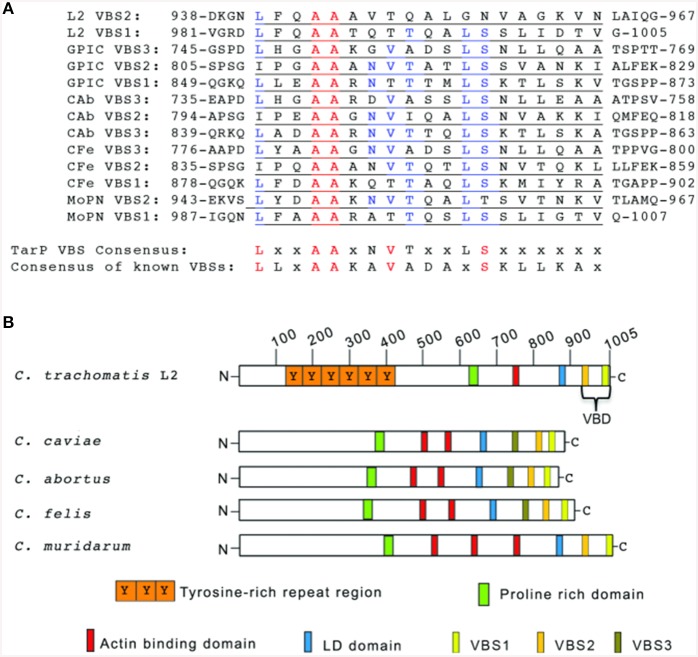
**TarP orthologs harbor multiple vinculin binding sites**. **(A)** Schematic of TarP orthologs from *C. trachomatis* serovar L2 (L2), *C. trachomatis* serovar D (D), *C. trachomatis* serovar A (A), *C. caviae* (GPIC), *C. abortus* (Cab), *C. felis* (CFe), and *C. muridarum* (MoPN). Indicated are the locations of the tyrosine-rich phosphorylation domain (orange box), the proline rich domain (green box), actin binding domains (red box), and the LD domain (blue box). Vinculin Binding Site 3 (VBS3; brown box), VBS2 (light orange box), and C-terminal VBS1 (yellow box) together comprise the Vinculin Binding Domain (VBD). **(B)** ClustalW sequence alignment of the putative VBS motifs. The numbers indicate the amino acid residue of the amino terminus or carboxy terminus. The consensus sequences shown are based on homology greater than 50%. A 19-residue consensus motif (LLxAAKAVADAxSKLLKAx) was generated by aligning by aligning the 11 VBSs of talin, the three VBSs of IpaA, and the two VBSs of sca4 for comparison to the TarP LD consensus (LxxAAxNVTxxLSxxxxxx). Identical amino acids are in red. Similar residues are in blue. “x” indicates any amino acid.

Some proteins that interact with vinculin, including the focal adhesion-associated talin, require VBS consisting of a 19-residue consensus motif (LLxAAKAVADAxSKLLKAx). This consensus sequence was determined from the alignment of the 11 VBSs of talin (Gingras et al., 2005), the three VBSs of IpaA (Park et al., 2011b) and the two VBSs of sca4 (Park et al., 2011a). We hypothesized that the robust vinculin recruitment may involve the virulence factor TarP due to its established function as a signaling scaffold (Lane et al., 2008). Through bioinformatics analyses, candidate VBS motifs were identified in the TarP orthologs of all chlamydial species investigated (Figure 2A). Together, these VBSs comprised the VBD, with VBS1 being the most C-terminal (Figure 2B).

### *C. caviae* TarP is sufficient to recruit vinculin

**Figure 3:**
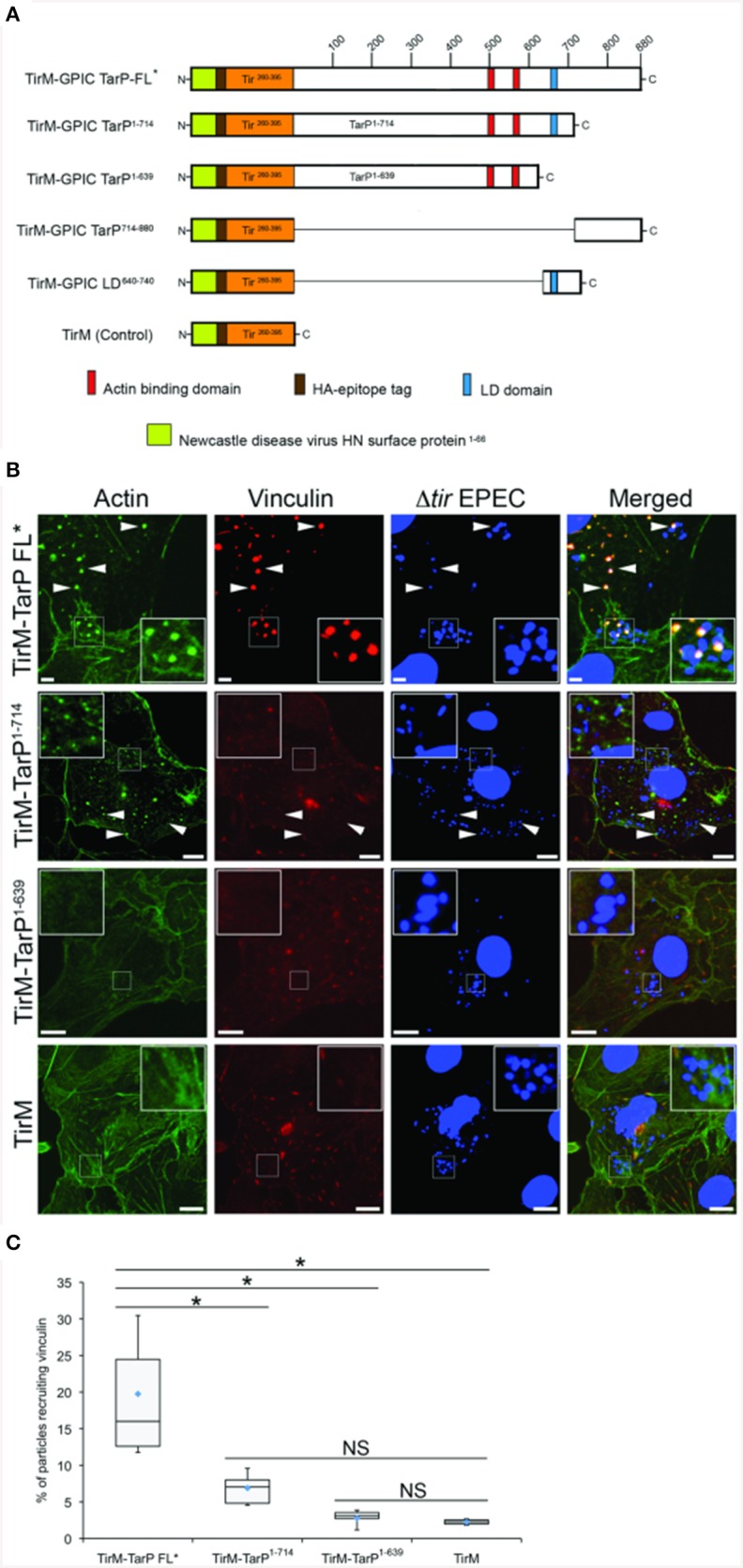
*****C. caviae*** TarP-mediated vinculin recruitment requires the vinculin binding domain**. **(A)** Schematic indicating the locations of the membrane targeting sequence (yellow box), Ha-tag (brown box), TirM (amino acids 260–395; orange box), actin binding domains (red box), and the LD domains (blue box). The numbers indicate amino acid positions encoded within the *C. caviae tarP* gene. **(B)** Cos7 cells transfected with plasmids encoding full length TarP (TirM-TarP FL), progressive TarP deletion derivatives (TirM-TarP1-714 or TirM-TarP-1-639), or the TirM control were infected with Δ*tir* EPEC to induce clustering of the fusion protein. Transfected cells were identified by their ability to bind Δ*tir* EPEC. The white arrowheads indicate colocalization of vinculin (red) with Δ*tir* EPEC (blue). Vinculin was visualized with an anti-vinculin antibody. Bacteria were visualized by DAPI staining. Scale bars: 5 μm. Refer to Figures 4, **6A** for schematic of TarP and its deletion derivatives. **(C)** Adhered EPEC able to recruit vinculin were enumerated for TirM, TirM-TarP-1-639, TirM-TarP1-714 and TirM-FL-TarP^*^, and data represented as box and whisker plot. Data compiled from three independent experiments. Plot shows range (statistical outliers excluded), first and third quartiles, and overall median (horizontal line). Diamonds show means. A range of 360–600 particles was counted. Insets show a magnification of a selected area of the cell. The asterisk and bars indicate significance difference between specific groups (One-way ANOVA, Tukey's *post-hoc* test, *P* < 0.00001). NS, not significant.

We tested the putative TarP VBD for its ability to recruit vinculin to the plasma membrane using a previously described assay based on the clustering of ectopically expressed and plasma membrane-targeted TarP fused to the extracellular domain of the Tir protein of enteropathogenic *Escherichia coli* (EPEC) (Thwaites et al., 2014). Briefly, the TarP domain of interest (Figure 3A) was fused to the extracellular and transmembrane domains of Tir. When transiently expressed in host cells, the Tir-TarP fusions (TirM-TarP) were targeted to the plasma membrane where they are clustered by adhered Δ*tir* EPEC to initiate signaling (Campellone et al., 2004; Thwaites et al., 2014). Through deletion analysis of TarP, the heterologous EPEC-based system confirmed the role of VBD (TirM-TarP^714−880^) in vinculin recruitment. In Figure 3B, vinculin recruitment was induced by full-length TarP (TirM-TarP FL). Removing the C-terminal 166 amino acid residues led to the loss of vinculin recruitment, indicating that this region fully accounted for the vinculin recruitment activity of full-length TarP. Scoring individual EPEC particles for colocalization with vinculin and expressing data as recruitment frequency revealed an increase of three-fold for TarP-FL relative to TirM-TarP^1−714^ (Figure 3C; *p* < 0.005; ANOVA and Tukey-Kramer *post-hoc* test). Taken together, we concluded that TarP itself could recruit vinculin, and we attribute this activity to the putative VBD motif located within the last 166 amino acids.

**Figure 4:**
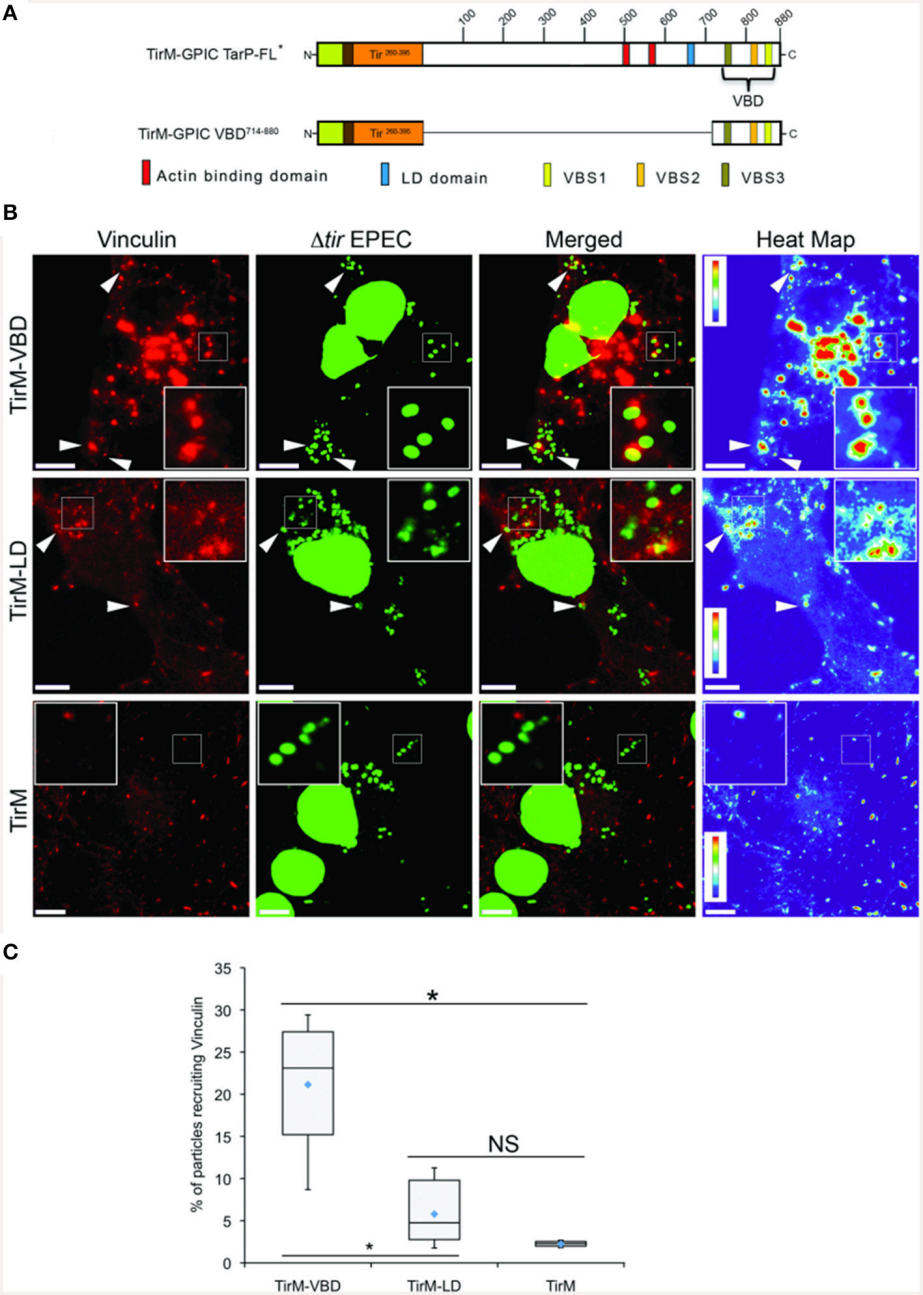
**The vinculin binding domain of TarP is functional and able to recruit vinculin**. **(A)** Schematic of full length *C. caviae* TarP deleted of the proline rich domain (TarP FL^*^) and the TarP Vinculin Binding Domain (VBD; TirM-VBD) indicating the locations of the N-terminal membrane targeting sequence (yellow box), Ha-tag (brown box), TirM (amino acids 260–395; orange box), actin binding domains (red box), the LD domains (blue box), Vinculin Binding Site 3 (VBS; brown box), VBS2 (light orange box), and C-terminal VBS1 (yellow box). The numbers indicate amino acid positions encoded within the *C. caviae* TarP gene. ^*^Denotes TarP derivative deleted for the PRD. **(B)** Cos7 cells transfected with plasmids encoding the VBD (TirM-VBD), the LD domain (TirM-LD), or the TirM control were infected with Δtir EPEC to induce clustering of the fusion protein. Transfected cells were identified by their ability to bind Δ*tir* EPEC. Heat map of the vinculin channel highlights the intensity of vinculin recruited to sites of bacterial adherence. The white arrowheads indicate colocalization of vinculin (red) with Δ*tir* EPEC (false-colored green). Vinculin was visualized with an anti-vinculin antibody. Bacteria were visualized by DAPI staining. Scale bars: 10 μm. Heat map representation (red, high; violet, low) was generated using imageJ. **(C)** Adhered EPEC able to recruit vinculin were enumerated for TirM, TirM-LD and TirM_VBD, and data represented as box and whisker plot. Data compiled from three independent experiments. Plot shows range (statistical outliers excluded), first and third quartiles, and overall median (horizontal line). Diamonds show means. A range of 310–680 particles was counted. Insets show a magnification of a selected area of the cell. The asterisk and bars indicate significance difference between specific groups (One-way ANOVA, Tukey's *post-hoc* test, *P* < 0.00001). NS, not significant.

Interestingly, vinculin was not recruited by the FAK-interacting LD domain present in TirM-TarP^1−714^, despite the known role of this protein in the FAK signaling pathway in focal adhesions. To confirm this observation, TirM-VBD and TirM-LD (Figure 4A) were evaluated for vinculin recruitment in transiently transfected Cos7 cells. Fusion proteins localized to the plasma membrane were clustered by EPEC Δ*tir* mutants adhered to the cell surface. After 1 h, the samples were fixed for staining with antibody to vinculin. Images were acquired and the frequency of vinculin recruitment was determined for each sample. In Figure 4B, there was a noticeably decreased incidence of vinculin recruitment in the TirM-LD sample compared to TirM-VBD. Quantification of recruitment incidence revealed that TirM-VBD was statistically significant different from the negative control TirM, in contrast to TirM-LD.

### The VBD domain requires vinculin to recruit actin

**Figure 5:**
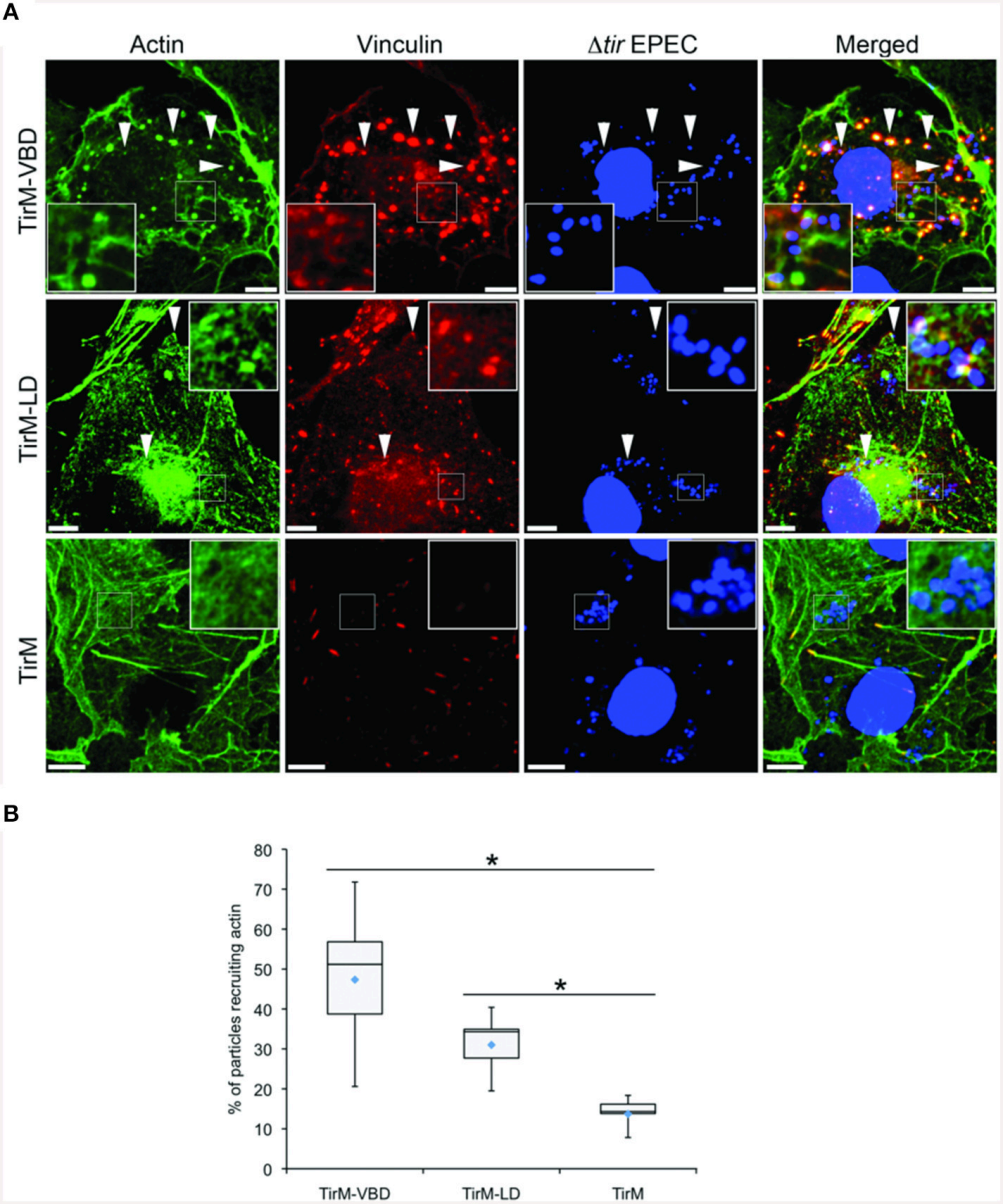
**The vinculin binding domain mediates the recruitment of both vinculin and actin**. **(A)** Cos7 cells transfected with the plasmid encoding the TarP Vinculin Binding Domain (TirM-VBD), the LD domain (TirM-LD), or the control vector TirM were infected with Δ*tir* EPEC to induce clustering of the proteins. Transfected cells were identified by staining for the HA-tag present in the fusion proteins. The white arrowheads indicate colocalization of actin and vinculin with adhered Δ*tir* EPEC. Filamentous actin (green) and vinculin (red) were visualized with phalloidin or an anti-vinculin antibody, respectively. Bacteria (blue) were visualized by DAPI. Scale bars: 10 μm. **(B)** Adhered EPEC able to recruit actin were enumerated and data represented as box and whisker plot. Data compiled from three independent experiments. Plot shows range, first and third quartiles, and overall median (horizontal line). Diamonds indicate means. A range of 600–730 particles was counted. Insets show a magnification of a selected area of the cell. The asterisk and bars indicate significance difference between specific groups (One-way ANOVA, Tukey's *post-hoc* test, *P* < 0.00001).

Having established the role of VBD in vinculin recruitment, we next addressed the functionality of this interaction in regards to actin recruitment and filamentous actin (F-actin) assembly. The ability of the VBD domain to recruit actin was compared to the LD motif, which was previously shown to induce a FAK-dependent signaling to mediate actin recruitment, and thus was the positive control. Using the EPEC-based assay, plasma membrane-localized TirM-VBD, TirM-LD, or TirM was clustered by the adhered EPEC to induce signaling. Recruitment of actin and vinculin was assessed for each sample by staining with phalloidin and anti-vinculin antibody, respectively. As shown in Figure 5A, TirM-VBD and TirM-LD were able to recruit actin, but only the former efficiently localized vinculin underneath the adhered EPEC bacteria. The negative control TirM recruited neither actin nor vinculin. Incidence of actin recruitment was quantified for each sample (Figure 5B). Incidences for both TirM-VBD andTirM-LD were statistically significantly different than that for the negative control. Based on these data, we conclude that the VBD domain is competent in signaling to recruit actin.

**Figure 6:**
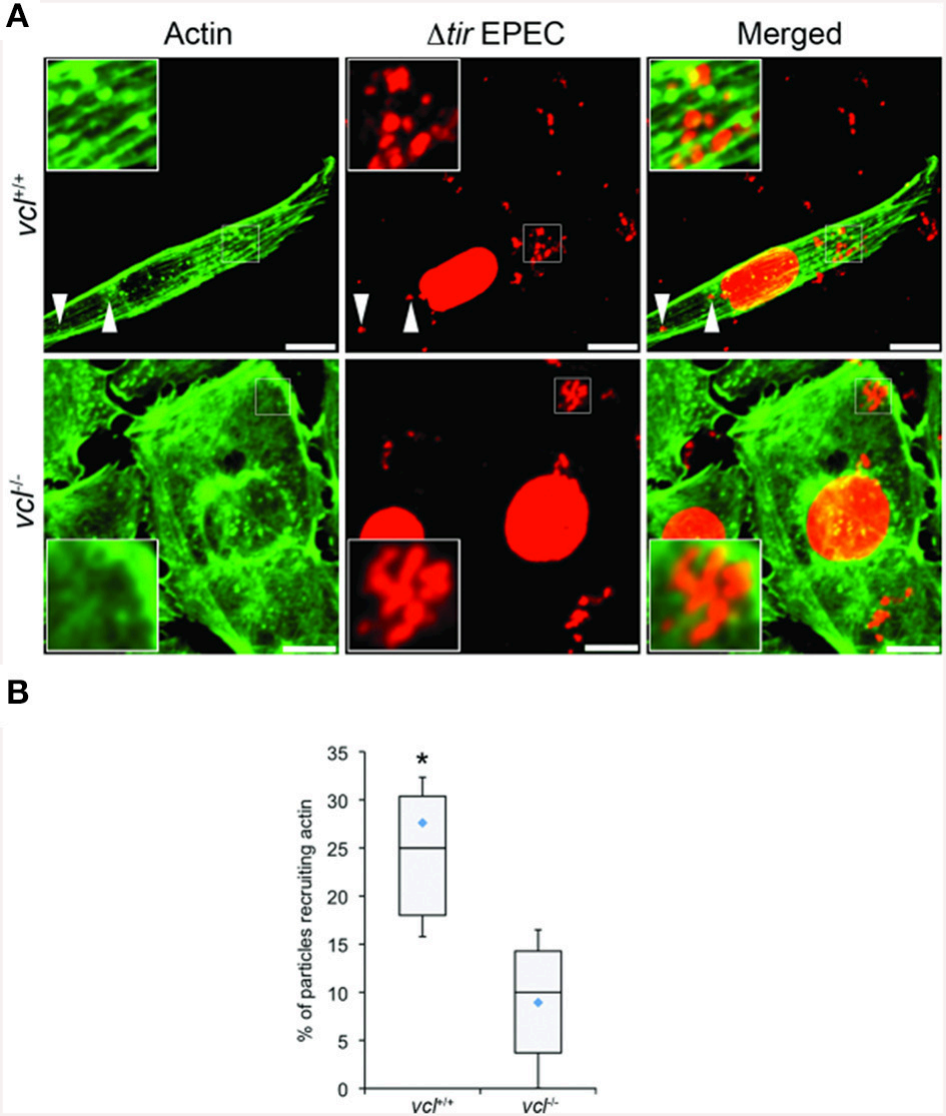
**The ability of the vinculin binding domain to recruit actin is dependent on vinculin**. **(A)** *vcl*^+∕+^ and *vcl*^−∕−^ mouse embryo fibroblasts (MEFs) expressing the Vinculin Binding Domain (TirM-VBD) were infected with Δ*tir* EPEC, and the recruitment of actin monitored. Actin (green) or bacteria (false-colored red) were visualized with phalloidin or DAPI, respectively. The white arrowheads indicate colocalization of actin with Δ*tir* EPEC. Scale bars: 10 μm. **(B)** Adhered EPEC colocalizing with actin in *vcl*^+∕+^ and *vcl*^−∕−^ MEFs were enumerated and data represented as box and whisker plot. Data compiled from three independent experiments. Plot shows range (statistical outliers excluded), first and third quartiles, and overall median (horizontal line). Diamonds show means. A range of 170–235 particles was counted per sample. Insets show a magnification of a selected area of the cell. Asterisk indicates statistical significance (One-way ANOVA, Tukey's *post-hoc* test, *P* < 0.00001).

We tested directly the dependence of VBD-mediated actin recruitment on vinculin using *vcl*^−∕−^ MEFs. Using the same EPEC-based assay in conjunction with TirM-VBD, the transfected fibroblasts were evaluated for actin recruitment at the base of the adhered EPEC. The contrasting cell morphologies of *vcl*^+∕+^ and *vcl*^−∕−^ MEFs were expected and consistent with previous reports. Analysis of the confocal images revealed the essential role of vinculin in VBD-mediated actin recruitment (Figure 6A). Punctate F-actin aggregates could be observed below the adhered EPEC in WT MEFs. In contrast diffuse phalloidin staining were seen in the knockout cells. Quantification of the incidence of actin recruitment confirmed the role of vinculin. The frequencies of actin recruitment were significantly greater (>2.5-fold) relative to the *vcl*^−∕−^ samples (Figure 6B; *p* < 0.005; ANOVA and Tukey-Kramer *post-hoc* test).

### Functional analysis of VBD deletion derivatives reveals an important role for VBS1 in vinculin recruitment

**Figure 7:**
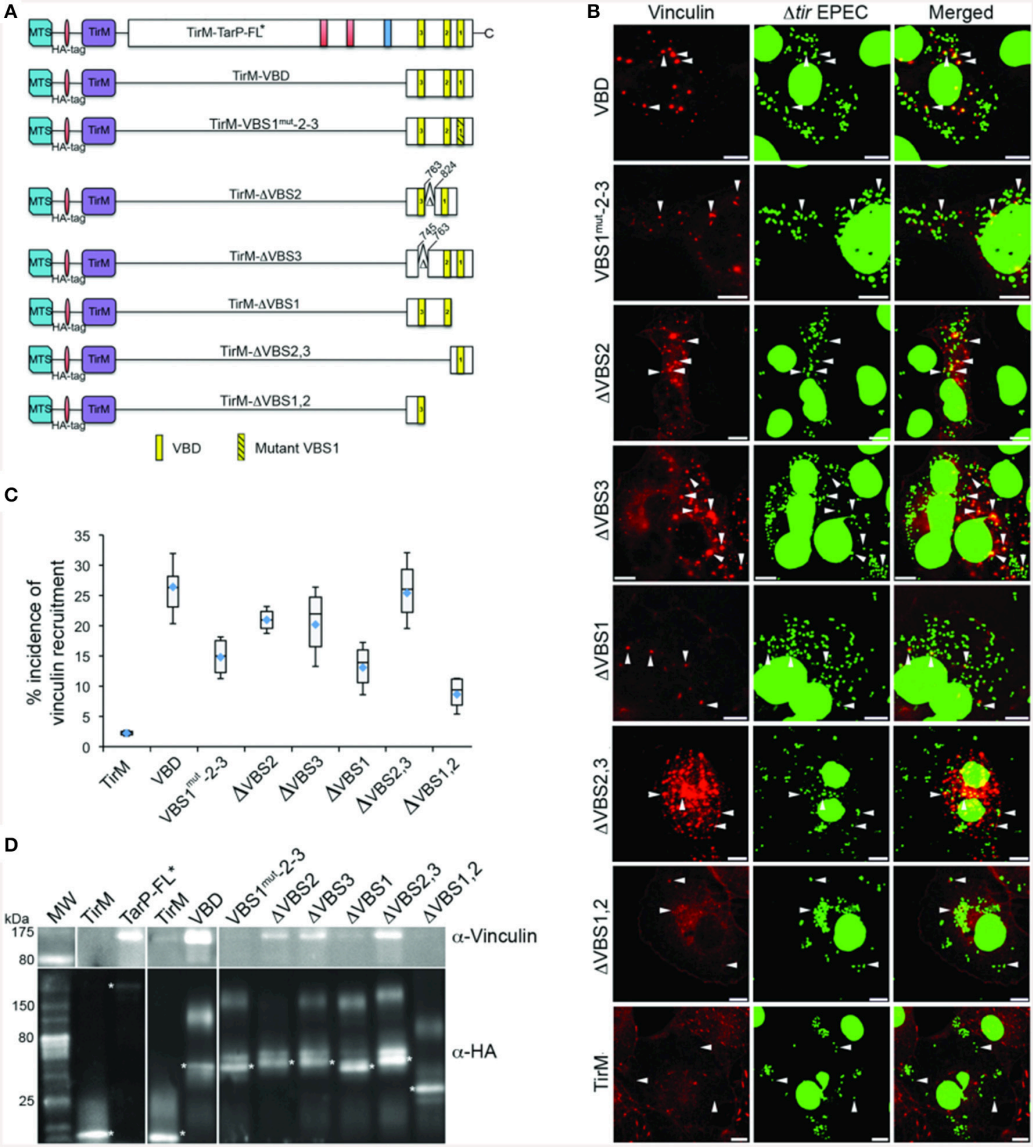
**Functional evaluation of the individual VBS motif revealed the importance of VBS1**. **(A)** Schematic of Vinculin Binding Domain (TirM-VBD) derivatives designed for use with the EPEC-based assay. Indicated are the locations of the N-terminal membrane targeting sequence (MTS; cyan box), Ha-tag (red box), TirM (amino acids 260–395; purple box), proline rich domain (green box), actin binding domains (red box), the LD domains (blue box), Vinculin Binding Site 3 (VBS; brown box), VBS2 (orange box), and C-terminal VBS1 (yellow box). Δ indicates amino acids deleted in mutant TarP proteins, and the numbers indicate amino acid positions encoded within the *C. caviae* TarP gene. Yellow box with black stripes indicates a mutated VBS derivative. **(B)** Cos7 cells transfected with plasmids encoding the VBD (TirM-VBD), a VBD mutant derivative in which the critical leucines of VBS1 were converted to serines [TirM-VBS1(mut)-2-3], the VBD deleted for VBS1 (TirM-ΔVBS1), the VBD deleted for VBS2 (TirM-ΔVBS2), the VBD deleted for VBS3 (TirM-ΔVBS3), VBS1 or VBS3 were infected with Δ*tir* EPEC to induce clustering of the fusion protein. Transfected cells were identified by their ability to bind Δ*tir* EPEC. The white arrowheads indicate colocalization of Vinculin (red) with Δ*tir* EPEC (false-colored green). Vinculin was visualized with an anti-Vinculin antibody. Bacteria were visualized by DAPI staining. Scale bars: 10 μm. **(C)** Adhered EPEC able to recruit Vinculin were enumerated for VBD, VBS1(mut)-2-3, ΔVBS1, ΔVBS2, ΔVBS3, VBS1, or VBS3, and data represented as box and whisker plot. Data compiled from three independent experiments. The graph shows range (statistical outliers excluded), first and third quartiles, and overall median (horizontal line). Diamonds indicate means. A range of 200–550 particles was counted. The asterisk and bars indicate significant difference between specific groups (One-way ANOVA, Tukey's *post-hoc* test, *P* < 0.05). **(D)** The interaction of vinculin with the VBSs was evaluated by co-immunoprecipitation of HA-tagged VBD derivatives along with the full-length TarP and the negative control TirM at 32 h post-transfection. The co-precipitated vinculin protein and the recombinant TarP and/or VBD constructs were visualized by western blot using antibodies to vinculin and the HA-tag, respectively. The HA-tagged proteins are indicated by asterisks. Note that the loss of VBS1 either through deletion or mutation consistently led to the loss of interaction with vinculin.

The *S. flexneri* effector IpaA possesses three functional VBSs in tandem arrangement, of which the highest affinity C-terminal motif functioning as the trigger for vinculin activation (Tran Van Nhieu et al., 1997). Like the IpaA effector, *C. caviae* TarP harbors three VBS motifs. Helical wheel representations of the *C. caviae* TarP VBS motifs showed VBS1 to be most similar to the C-terminal VBS of IpaA (IpaA-VBS1), with comparable directions and magnitudes of the hydrophobic moments. Conversely, VBS2 or VBS3 have smaller magnitudes of the hydrophobic moments, indicating lower amphiphilicity (Figure S2). Because of the functional differences between the IpaA VBSs, we investigated if the same could be true for TarP VSB1, 2, and 3. Various combinations of VBSs were investigated for vinculin recruitment frequency in the context of the EPEC system (Figure 7A). As shown in Figures 7B,C, deletion of either VBS2 or VBS3 while retaining VBS1 (TirM-ΔVBS2, TirM-ΔVBS3, or TirM-ΔVBS2,3) had minimal influence on the incidence of vinculin recruitment in comparison to the WT VBD, hinting at the greater functional importance of VBS1. This was confirmed through the progressive deletions of the VBD that removed either VBS1 only or VBS1 and VBS2, with both deletion constructs leading to notable decreases in the incidence of vinculin recruitment. Scoring individual EPEC particles for colocalization with vinculin demonstrated that vinculin recruitment frequencies decreased 1.6 and 2.4-fold in cells expressing TirM-ΔVBS1 or TirM-ΔVBS1,2 respectively, relative to WT VBD (Figure 7C; *p* < 0.03; ANOVA and Tukey-Kramer *post-hoc* test). Also, a VBD mutant derivative (VBS1^mut^-2-3) in which the conserved hydrophobic residues of VBS1 were changed to serine was quantitatively and qualitatively similar to ΔVBS1. The data pointed to VBS1 accounting for the majority of VBD function.

A co-immunoprecipitation (co-IP) assay was conducted in parallel to the cell-based assays above. TarP-FL, the VBD and its derivatives were purified from transfected lysates by immunoprecipitation with an α-HA tag monoclonal antibody. Vinculin co-precipitation was monitored in the anti-HA pulldown samples. Sample loading was adjusted for the amount of the HA signal pulled down to account for expression and immunoprecipitation levels. An asterisk indicated the band for each fusion protein. As shown in Figure 7D, TarP-FL and VBD interacted with vinculin. Because of the overwhelming strength of the vinculin signal in both TarP-FL and VBD, it was necessary to limit the exposure to a shorter time than the rest of the blot. The co-IP studies also confirmed the predominant role of VBS1 in vinculin binding, as VBS1 deletion or mutation diminished the levels of vinculin pulled down. Interestingly, the respective levels of pulldown by TarP-FL and VBD were considerably greater than those derivatives retaining VBS1. This is in contrast to the set of data obtained by microscopy. This could be due to the different experimental systems, where the cell-based approaches monitored vinculin recruitment at the plasma membrane, whereas co-IP did not distinguish between differently localized TarP-vinculin complexes, some of which may have been cytosolic. In addition, the cell-based assay may have additional host cell factors that influenced the recruitment and retention of vinculin.

### The LD and VBD motifs function independently in recruiting FAK and vinculin, respectively

**Figure 8:**
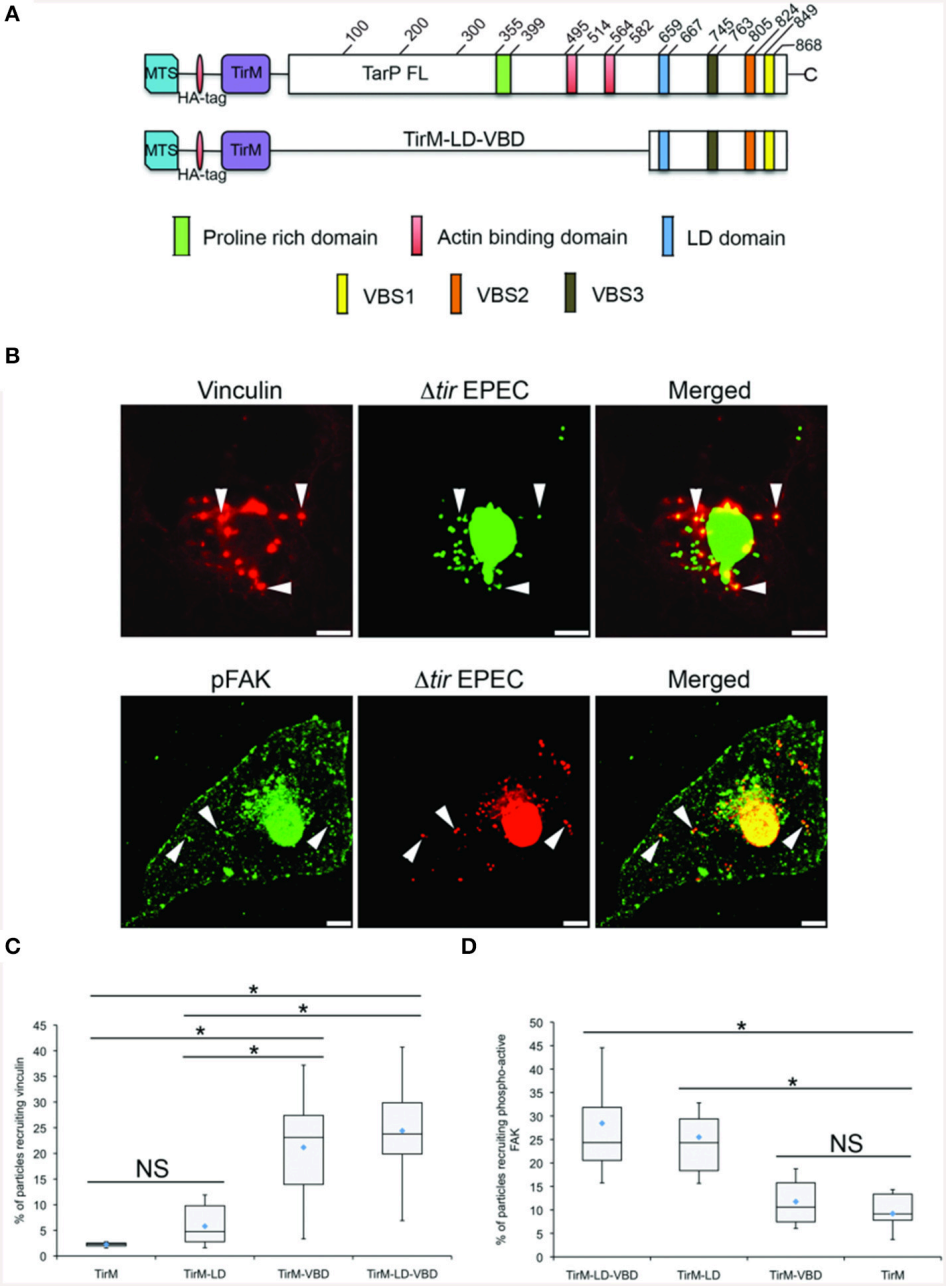
**The LD-VBD protein is functional and able to recruit phospho-active or vinculin**. **(A)** Schematic of full length TarP (TarP-FL) or its derivative, the LD-VBD, which represents a combination of the TarP LD domain and the TarP Vinculin Binding Domain (VBD). Please see Figure 4 legend for details. **(A,B)** Cos7 cells transfected with the plasmid encoding LD-VBD were infected with Δ*tir* EPEC to induce clustering of the proteins. Transfected cells were identified by the capacity to facilitate bacteria attachment. The white arrowheads indicate colocalization of phospho-active FAK (pFAK) or vinculin with adhered Δ*tir* EPEC. pFAK (green) and vinculin (red) were visualized with an anti-FAK (phospho Y397) or an anti-vinculin antibody, respectively. Bacteria [false-colored green (vinculin) or red (pFAK)] were visualized by DAPI. Scale bars: 10 μm. **(C,D)** Adhered EPEC able to recruit vinculin **(C)** or pFAK **(D)** were enumerated and data represented as box and whisker plot. Data compiled from two independent experiments. Plot shows range (statistical outliers excluded), first and third quartiles, and overall median (horizontal line). Diamonds show means. Roughly 200 particles were counted for both conditions. The asterisk and bars indicate significance difference between specific groups (One-way ANOVA, Tukey's *post-hoc* test, *P* < 0.00001). NS; not significant. For comparison, previously quantified data for the LD domain alone or the VBD domain alone with pFAK or vinculin was included.

In the context of TarP, the VBD represents a further contribution to the growing list of functional domains that subvert the host actin cytoskeleton. One of these is the LD motif, which overlaps with the previously characterized TarP F-actin binding domain (FAB1). This motif mediated a FAK and Arp2/3 complex-dependent actin remodeling (Thwaites et al., 2014). Its close proximity to VBD hinted at a potential interaction between the two domains. Using the EPEC system, the functions of VBD and LD were investigated by expressing the C-terminal 241 amino acid residues of TarP spanning both motifs (TirM-LD-VBD) (Figure 8A). The recruitment of either Tyr397-phosphorylated FAK (pY397-FAK) or vinculin was monitored in parallel with TirM-LD and TirM-VBD (Figure 8B). We reasoned that an additive or synergistic interaction would result in the increased incidence of recruitment of pY397-FAK and vinculin to the clusters of TirM-LD-VBD at the plasma membrane. What we observed was that the incidences of recruitment of vinculin or pY397-FAK by TirM-VBD were similar to those of either the LD or the VBD domain alone (Figures 8C,D), indicating that vinculin recruitment by the VBD motif did not interfere with the recruitment of FAK by the LD domain, and vice versa. There was neither mutual inhibition nor additive/synergistic interactions.

**Figure 9:**
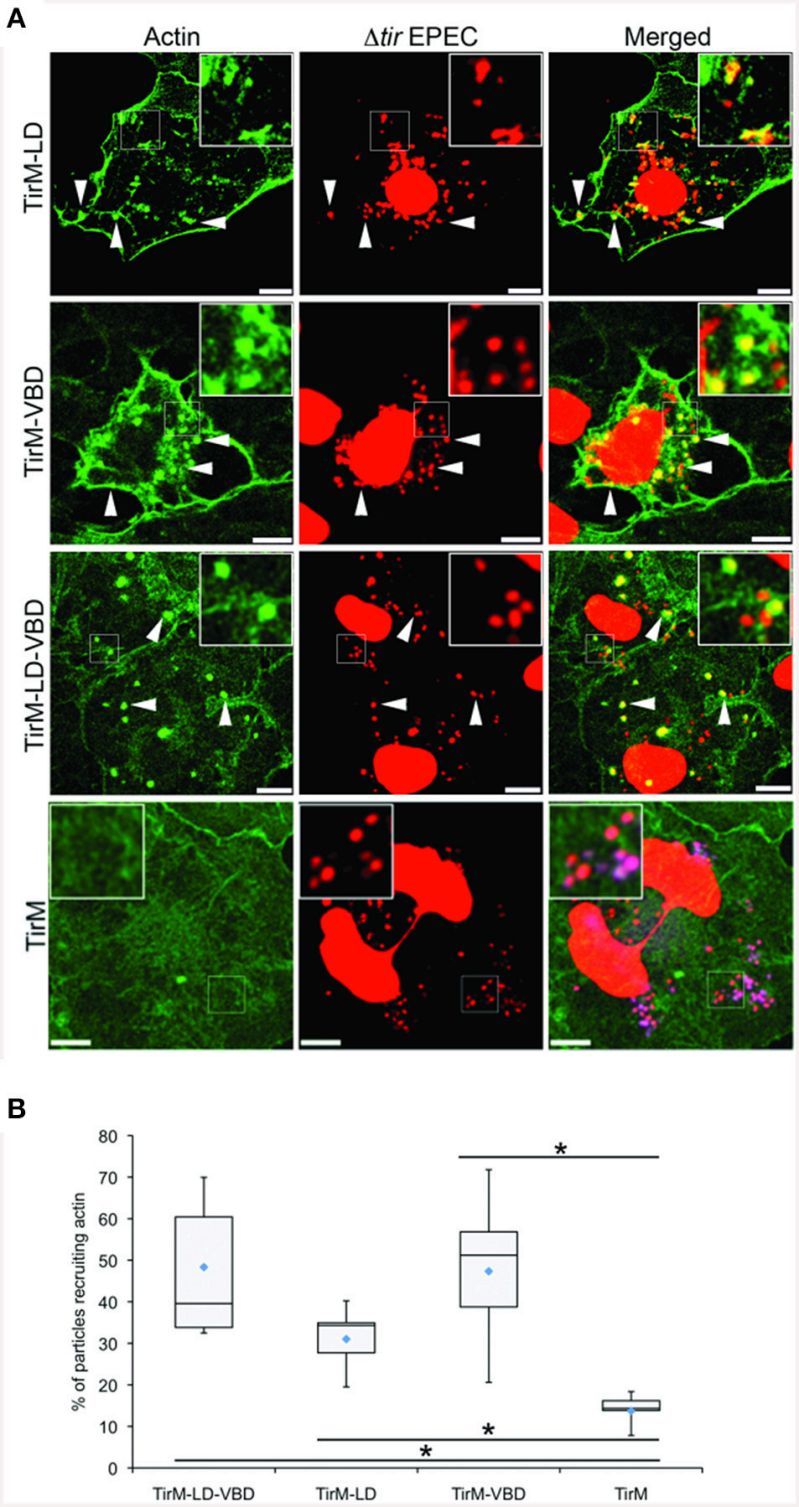
**The LD-VBD induces robust actin recruitment**. **(A)** Cos7 cells transfected with the plasmid encoding LD-VBD were infected with Δ*tir* EPEC to induce clustering of the proteins. Transfected cells were identified by the capacity to facilitate bacteria attachment. The white arrowheads indicate colocalization of actin with adhered Δ*tir* EPEC. Actin (green) and bacteria (false-colored red) were visualized with phalloidin or DAPI, respectively. Scale bars: 10 μm. **(B)** Adhered EPEC able to recruit actin were enumerated and data represented as box and whisker plot. Data compiled from three independent experiments. Plot shows range (statistical outliers excluded), first and third quartiles, and overall median (horizontal line). Diamonds indicate means. A range of 300–680 particles were counted. The asterisk and bars indicate significance difference between specific groups (One-way ANOVA, Tukey's *post-hoc* test, *P* < 0.00001). For comparison, previously quantified data for the LD domain alone or the VBD domain alone actin was included.

The LD, VBD, and LD-VBD domains were evaluated for actin recruitment using the EPEC assay by staining the samples with phalloidin and DAPI to visualize filamentous actin and adhered EPEC bacteria on the cell surface, respectively (Figure 9A). Incidences of recruitment were monitored in a single-blind experiment, and data was expressed as a box and whisker plot (Figure 9B). TirM-LD-VBD was able to recruit actin at a frequency similar to that obtained for TirM-VBD. The value obtained for TirM-LD was consistent with our previous report (Thwaites et al., 2014). Based on the data, the FAK/LD and VBD/vinculin pathways do not exhibit an additive or synergistic interactions. It was not feasible to distinguish the relative contributions of each to the LD-VBD-mediated actin recruitment, and we could not exclude the possibility that signaling from the VBD inhibited that from the LD domain. We deem this unlikely, as FAK recruitment by the LD motif was not inhibited when VBD and vinculin were present.

## Discussion

Vinculin is exploited by a number of microbial pathogens as a means to productive infection. *S. flexneri, Rickettsia*, and enteropathogenic *E. coli* have all been reported to subvert vinculin functions directly through type III-secreted effectors (*S. flexneri* and EPEC) (Freeman et al., 2000; Park et al., 2011b) or cell surface antigens (*Rickettsia*) (Park et al., 2011a). In each case, vinculin recruitment is regarded as a mechanism to induce localized actin remodeling. Here, we show that vinculin is a cellular target for the invasion-associated effector TarP, and is required for chlamydial entry. Our results reveal that *Chlamydia*e have engineered VBS that share a remarkable level of homology with the VBSs of talin. It is now clear that *Chlamydia* employ a number of strategies to hijack actin polymerization, with evidence pointing toward mimicry as the prevailing theme.

An important component of focal adhesion structures, vinculin functions to anchor the actin cytoskeleton to the plasma membrane via the b1 cytoplasmic domain of integrins. Binding of the tail domain to F-actin requires that vinculin adopts an activated “open conformation.” Recently, the VBSs of talin have been reported to trigger vinculin activation by binding to its head domain and displacing interactions with tail domain (Bois et al., 2006). The binding of the Sca4 or IpaA VBSs to the head domain can displace the intramolecular interactions with the tail domain to facilitate vinculin activation and, ultimately, actin recruitment (Tran Van Nhieu et al., 1997; Hamiaux et al., 2006; Park et al., 2011a,b). Importantly, this scenario bypasses the “pre-activation” steps described for talin-mediated vinculin activation (Fillingham et al., 2005; del Rio et al., 2009; Grashoff et al., 2010), allowing these effectors to efficiently sequester this F-actin binding protein for pathogenic purposes. Interestingly, the recruitment of vinculin observed during *Chlamydia* infection was due to a C-terminal VBD within TarP. *Chlamydia* TarP orthologs harbor variable numbers of VBS motifs that together comprise the VBD. Like IpaA, the VBD of *C. caviae* TarP consists of three VBS motifs and functions to recruit actin in a vinculin-dependent fashion. The high local concentration of these VBS motifs would ensure a tight association with vinculin. Indeed, such may be the case for *S. flexneri*. Izard et al. proposed that IpaA alters vinculin function through its high-affinity interaction between its VBS1 and the vinculin head domain (Izard et al., 2006). It was proposed that this interaction bypasses the force generation requirement to mechanically stretch and activate vinculin.

Interestingly, TarP harbors three VBSs with variations in amino acid residues in the hydrophobic face of the amphipathic α-helices that likely manifest as differences in the strength of interaction with vinculin. Izard et al. reported that there are at least two residues of the IpaA-VBS1 consensus, Tyr-2 and Ala-4, which contribute to very high-affinity interactions with the hydrophobic interface with vinculin (Izard et al., 2006). For the TarP VBS motifs, it is clear that there is a perfect conservation of the alanine residue at position 4 and considerable variation at position 2. The three VBSs of IpaA appear to have different roles in *Shigella* entry, which has been traced to differences in affinity for vinculin (reviewed in Carayol and Tran Van Nhieu, 2013). The most carboxy-terminal VBS1 promotes vinculin activation by functioning as a supermimic of talin. Coincidentally, the most C-terminal VBS of TarP (VBS1), which most closely resembled the IpaA VBS1 (Figure S2), demonstrated the highest level of vinculin recruitment and was critical for vinculin interaction in pulldown experiments. Thus, it appears that the configuration of TarP VBSs reflects that found in IpaA. It would be of interest to investigate in greater detail the functions of TarP VBS2 and VBS3, which may include stabilization and maturation of the protein complex to maintain vinculin activation and actin binding.

The dependence of actin recruitment on vinculin contrasts with the findings of Jiwani et al. which showed a direct F-actin binding activity of TarP via the newly discovered FAB1 and FAB2 domains, the latter overlapping with *C. caviae* GPIC VBS3 (Jiwani et al., 2013). It was clear both from image acquisition and quantification of the level of actin recruitment that the VBD did not efficiently recruit actin to the plasma membrane in *vcl*^−∕−^ cells. If the TarP VBD had F-actin binding activity in the context of cultured cells, actin should have been present. We showed in a separate study that FAB1, which overlaps with the LD domain, recruits actin in a manner dependent on FAK (Thwaites et al., 2014). This discrepancy could be explained by the different experimental systems used. Whereas, Jiwani et al. used purified TarP derivatives to interrogate F-actin binding *in vitro*, we relied on ectopic expression in cells and monitored the recruitment of endogenous vinculin and actin. It is important to emphasize that, whilst interaction with vinculin may be important in invasion, we cannot discount the possibility of post-invasion functions for FAB1 and FAB2.

Interestingly, the duration of vinculin localization to the sites of invasion was longer than that of actin. At 120 min, which is the latest time point studied, vinculin remained present. This is in contrast to the transient recruitment of actin, which we reported to be less than 5 min (Carabeo et al., 2002). It is possible that after the termination of signaling, vinculin remained associated with TarP. It is intriguing to speculate that this persistent interaction is significant in post-invasion stages of infection.

From a mechanistic point of view, it remains unclear how vinculin mediates chlamydia invasion. Our observations that FAK (Thwaites et al., 2014), vinculin (this study), talin, and paxillin (unpublished observations) were recruited to the sites of chlamydia invasion imply the formation of pseudo-focal adhesion structures that can organize the actin network to enable bacterial entry. A similar model has been proposed for Shigella (Tran Van Nhieu et al., 1997; Park et al., 2011b). This would imply that *Chlamydia* invasion is regulated to some extent by mechanisms similar to those that determine focal adhesion dynamics. Indeed, siRNA screens for host molecules necessary for *Chlamydia* infection have identified components of focal adhesion signaling, including DOCK180 (Elwell et al., 2008; Gurumurthy et al., 2010). Furthermore, the EB-associated type III effector TepP has been reported to interact with CrkI-II, which has a well-characterized role in FAK signaling (Zouq et al., 2009; Chen et al., 2014) further supporting a pseudo-focal adhesion signaling model. The relatively detailed knowledge of focal adhesion signaling may serve as a good framework for the characterization of FAK- and vinculin-dependent functions of TarP. An intriguing model implicates TarP in a role similar to that of integrin molecules, serving as a molecular scaffold to which focal adhesion-associated proteins bind and initiate signaling to and interaction with the host actin cytoskeleton.

Here, we addressed the molecular basis of vinculin recruitment at the adhesion sites, its role in actin recruitment, and in the uptake of *Chlamydia* EBs. During the course of this study, we also investigated the potential functional interaction between the LD and VBD motifs. While the LD and VBD motifs did not exhibit signaling cooperation, it remains possible that these domains function with other motifs, such as the phosphodomain of *C. trachomatis*, and the actin-binding domains or with pathways involving host cell receptors. Multiple pathways of invasion have been reported for *Chlamydia*. When one pathway is ablated, there was a consistent lack of complete compensation, i.e., other endogenous pathways that remained functional did not restore fully invasion efficiencies, indicating that the invasion pathways available to *Chlamydia* are non-redundant. It is intriguing to speculate that this may relate to cellular/tissue tropism *in vivo*, such as interaction of the pathogen with non-phagocytic and phagocytic cells, or columnar vs. stratified squamous epithelia. In addition, while these domains have been shown to function individually *in vitro*, it is a very intriguing possibility that in different contexts, they may also exhibit cooperative interactions to ensure an efficient actin remodeling to facilitate *Chlamydia* invasion.

## Author contributions

TT, AP, TP, and RC conceived and designed the experiments. TT, AP, and TP performed the experiments and collected the data. TT, AP, TP, and RC analyzed the data. TT, AP, TP, and RC prepared the figures and wrote the manuscript.

### Conflict of interest statement

The authors declare that the research was conducted in the absence of any commercial or financial relationships that could be construed as a potential conflict of interest.

## Supplementary material

Figure S1 **Chlamydia recruits vinculin to sites of infection**. *C. caviae* (GPIC) EBs were visualized with DAPI were added to Cos7 cells and the infection allowed to proceed for 0, 10, 30, 60, 90, or 120 min as described in the Materials and Methods Section. The samples were stained with an anti-vinculin antibody and DAPI to visualize the bacteria. *C. caviae* EBs (green); vinculin (red). Scale bars: 5 μm. White arrowheads indicate vinculin/GPIC EB colocalization.

Figure S2 **The VBS motifs of TarP adopt an amphipathic a-helical structure. (A)** Schematic of *C. caviae* GPIC TarP is shown indicating the positions and nomenclature of the VBS motifs. **(B)** ClustalW sequence alignment of the GPIC VBS motifs, the mutant derivative of VBS1 used in the context of TirM-VBS1(mut)-2-3 construct, and the C-terminal VBS1 from the *Shigella flexneri* effector IpaA. Identical amino acids within each alignment are in red. Similar residues are in blue. **(C)** Schiffer-Edmundson helical wheel diagrams depicting the 19 amino acids of VBS1, VBS2, VBS3, mutant VBS1, and IpaA VBS1 created with DNAstar program demonstrating the possible amphipathic α-helical conformation. Helical wheel projects the arrangement of amino acids, and residue numbers are counted from the amino terminus. Amino acids are represented by the single letter code and are colored based on their chemical properties. Hydrophobic (I, L, V, M: black; A, G, P: Green), hydrophilic (S, T: yellow; Y: blue; N: pink), and charged (D, E: red; K, R, H: light blue). The lines indicate bonds between amino acids. The arrow indicates the direction of the calculated hydrophobic moment, which is quantitative measure of amphipathicity.
