# Supplementary figures and images for: Vinculin Interacts with the Chlamydia Effector TarP Via a Tripartite Vinculin Binding Domain to Mediate Actin Recruitment and Assembly at the Plasma Membrane
Source: Front Cell Infect Microbiol. 2015 Nov 30;5:88. doi: 10.3389/fcimb.2015.00088 (PMC4663276; doi:10.3389/fcimb.2015.00088)

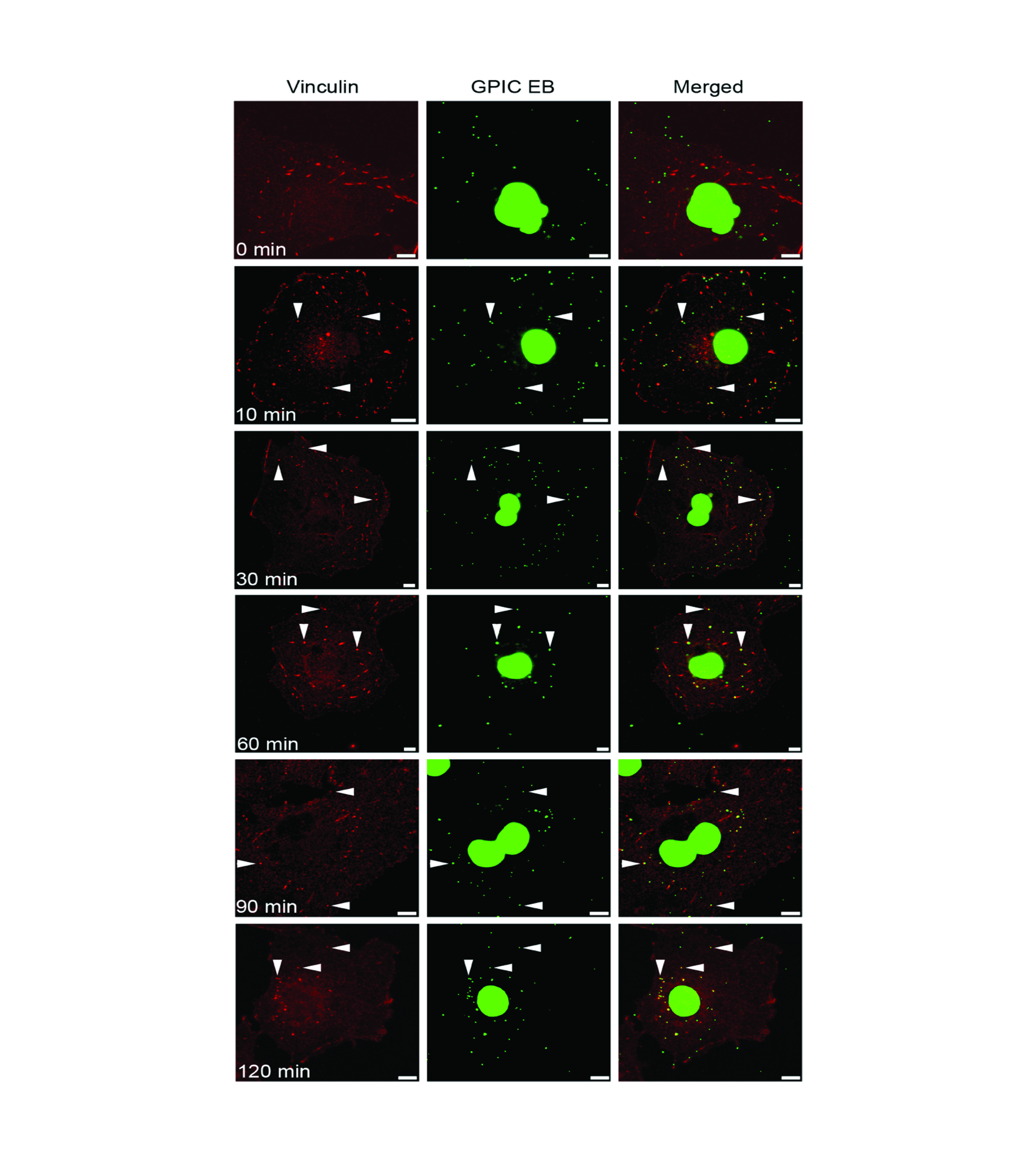

Supplement: Figure S1 — Chlamydia recruits vinculin to sites of infection. C. caviae (GPIC) EBs were visualized with DAPI were added to Cos7 cells and the infection allowed to proceed for 0, 10, 30, 60, 90, or 120 min as described in the Materials and Methods Section. The samples were stained with an anti-vinculin antibody and DAPI to visualize the bacteria. C. caviae EBs (green); vinculin (red). Scale bars: 5 μm. White arrowheads indicate vinculin/GPIC EB colocalization. [file Image1.TIF]

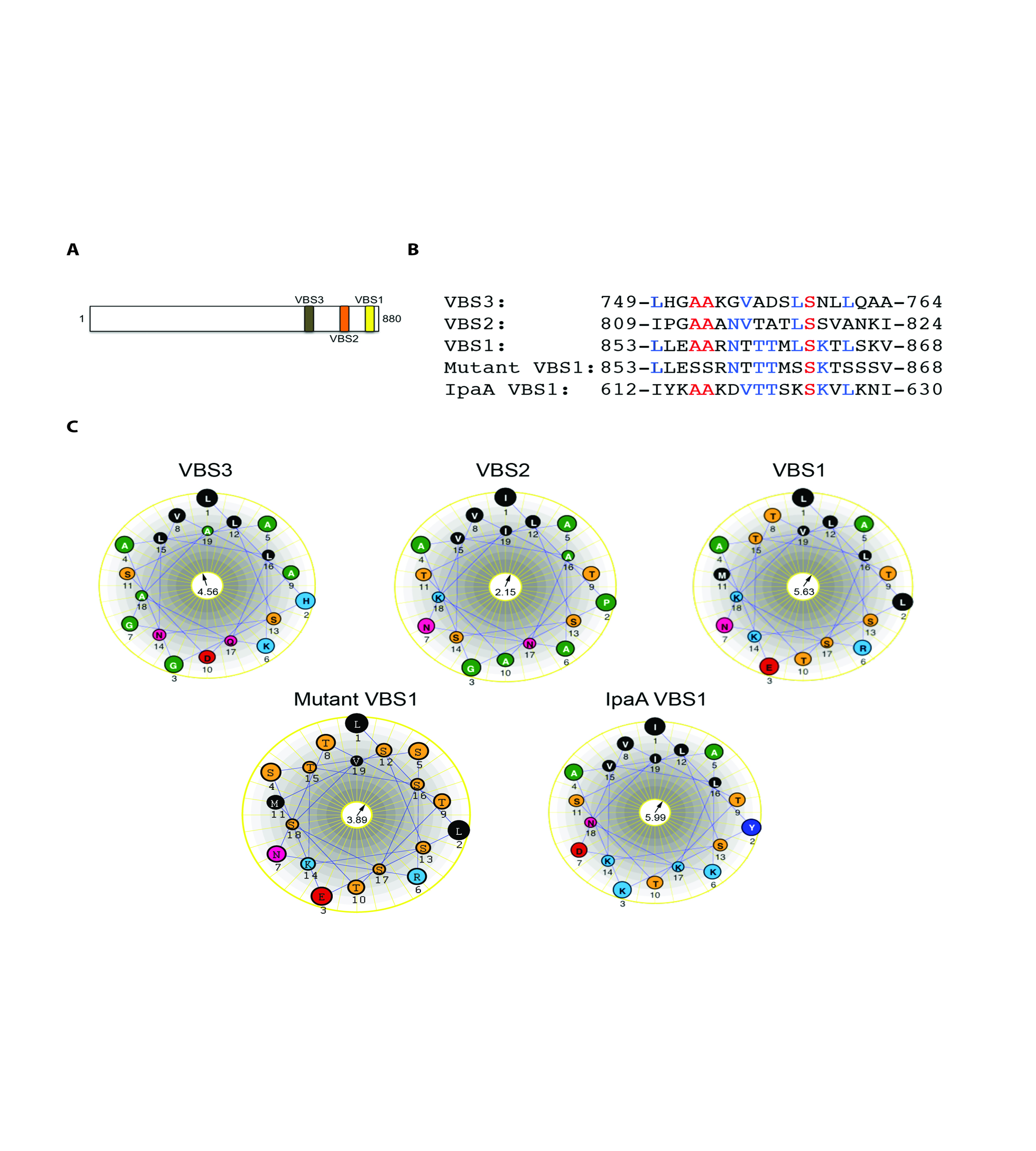

Supplement: Figure S2 — The VBS motifs of TarP adopt an amphipathic a-helical structure. (A) Schematic of C. caviae GPIC TarP is shown indicating the positions and nomenclature of the VBS motifs. (B) ClustalW sequence alignment of the GPIC VBS motifs, the mutant derivative of VBS1 used in the context of TirM-VBS1(mut)-2-3 construct, and the C-terminal VBS1 from the Shigella flexneri effector IpaA. Identical amino acids within each alignment are in red. Similar residues are in blue. (C) Schiffer-Edmundson helical wheel diagrams depicting the 19 amino acids of VBS1, VBS2, VBS3, mutant VBS1, and IpaA VBS1 created with DNAstar program demonstrating the possible amphipathic α-helical conformation. Helical wheel projects the arrangement of amino acids, and residue numbers are counted from the amino terminus. Amino acids are represented by the single letter code and are colored based on their chemical properties. Hydrophobic (I, L, V, M: black; A, G, P: Green), hydrophilic (S, T: yellow; Y: blue; N: pink), and charged (D, E: red; K, R, H: light blue). The lines indicate bonds between amino acids. The arrow indicates the direction of the calculated hydrophobic moment, which is quantitative measure of amphipathicity. [file Image2.TIF]
